# The Metabolism Symbiosis Between Pancreatic Cancer and Tumor Microenvironment

**DOI:** 10.3389/fonc.2021.759376

**Published:** 2021-12-16

**Authors:** Ying Li, Ju Zhang, Jie Xu, Shanglong Liu

**Affiliations:** ^1^ Department of Blood Transfusion, The Affiliated Hospital of Qingdao University, Qingdao, China; ^2^ Department of Operating Room, The Affiliated Hospital of Qingdao University, Qingdao, China; ^3^ Department of Nursing, Zaozhuang Second Health School, Zaozhuang, China; ^4^ Department of Gastrointestinal Surgery, The Affiliated Hospital of Qingdao University, Qingdao, China

**Keywords:** pancreatic cancer, tumor microenvironment, metabolism symbiosis, metabolic remodeling, crosstalk

## Abstract

Complex interactions occur between tumor cells and the tumor microenvironment. Studies have focused on the mechanism of metabolic symbiosis between tumors and the tumor microenvironment. During tumor development, the metabolic pattern undergoes significant changes, and the optimal metabolic mode of the tumor is selected on the basis of its individual environment. Tumor cells can adapt to a specific microenvironment through metabolic adjustment to achieve compatibility. In this study, the effects of tumor glucose metabolism, lipid metabolism, and amino acid metabolism on the tumor microenvironment and related mechanisms were reviewed. Selective targeting of tumor cell metabolic reprogramming is an attractive direction for tumor therapy. Understanding the mechanism of tumor metabolic adaptation and determining the metabolism symbiosis mechanism between tumor cells and the surrounding microenvironment may provide a new approach for treatment, which is of great significance for accelerating the development of targeted tumor metabolic drugs and administering individualized tumor metabolic therapy.

## Introduction

The composition of the tumor microenvironment in pancreatic cancer is complex and involves a dynamic process. A distinctive feature of malignant tumor pathology is the desmoplastic reaction, that is, the cancer cells are surrounded by a large number of dense fibrous matrix components. These matrix components lead tumors to show the characteristics of ischemia, and make it difficult for traditional chemotherapy drugs to enter tumor tissues. The enveloped tumor cells in microenvironment include various mesenchymal cells, a large amount of extracellular matrix extracellular matrix (ECM), and some soluble molecules such as cytokines, chemokines, and pro-angiogenic factors (1). Tumors form specific tumor microenvironments during their occurrence and development that are mainly divided into ecological microenvironments and physical microenvironments. The ecological microenvironment includes immune cells, fibroblasts, endothelial cells, and ECM. The physical microenvironment includes low oxygen, nutrient pressure, low pH, and oxidative pressure. There is an intricate relationship between the microenvironment and cells that plays an important role in tumor development, invasion and metastasis, chemotherapy resistance, tumor immunosuppression, and tumor cell metabolism remodeling (2).

In recent years, many studies have focused on the remodeling of tumor metabolism by the tumor microenvironment. Metabolic remodeling is one of the salient features of tumorigenesis and development, which can meet the rapid proliferation of tumor cells for energy and biological macromolecular substances. To maintain its malignant characteristics, tumors undergo significant changes in their metabolic patterns and pathways. This change is called metabolic remodeling (3). As early as the beginning of the 20^th^ century, Otto Warburg proposed that fast-proliferating cancer cells use “aerobic glycolysis” as their main energy production method. This metabolic pathway can help tumors adapt to the pancreatic cancer microenvironment, enhance their malignant biological behavior, and resistance to radiotherapy and chemotherapy. This process is accompanied by the accumulation of specific metabolites, such as glucose metabolites, lipid metabolites, and amino acids, which can regulate tumor-related signaling pathways through mechanisms such as competitive inhibition of epigenetic regulatory enzymes or post-translational modification of proteins. The pancreatic cancer microenvironment contains a large number of stromal cells, of which cancer-associated fibroblasts (CAFs) account for approximately half of the total number of cells in tumor tissues. Previous studies have shown that CAFs mainly promote tumor cell proliferation and metastasis by secreting large amounts of growth factors and chemokines (4). Furthermore, CAFs also undergo metabolic changes similar to tumor cells, from oxidative phosphorylation to aerobic glycolysis, thus producing and secreting metabolic intermediates such as lactic acid and ketone bodies, and these metabolic intermediates can be directly taken up by tumor cells to promote rapid proliferation (5). Demircioglu et al. reported that loss of focal adhesion kinase (FAK) in a subpopulation of CAFs causes the upregulation of Ccl6, Ccl11, Ccl12 and pentraxin-3 resulting in the enhancement of glycolysis in pancreatic cancers. FAK depletion in CAFs activate protein kinase A and lead to enhanced malignant cell glycolysis *via* CCR1/CCR2 on cancer cells (6). Moreover, it is demonstrated that as regulator of glutamate, glutamine, and cytokine release, Netrin G1 (NetG1) in CAFs and Netrin G1 Ligand (NGL-1) in pancreatic cancer cells enhanced tumorigenesis by allowing cancer cells to survive in low nutrient conditions and reduced death induced by NK cells (7). Limitation of nutrient availability is overcome partly by exchange of metabolites and cytokines between the stromal and cancer cells. Data shows that as the main matrix component in tumor tissues, CAFs not only promote tumor progression, but also directly supply the biomass needed for tumor cell synthesis and metabolism by secreting metabolic intermediate products (8). Metabolic crosstalk with stromal cells in the tumor microenvironment is one of important alternative sources of nutrient acquisition for pancreatic cancer. However, little is known about the molecular mechanism of this metabolic change, and the metabolic relationship between pancreatic cancer tumor cells and the tumor microenvironment remains unclear. This article mainly focuses on the metabolic symbiosis and critical metabolites in the microenvironment of pancreatic cancer, and discusses the mechanism of metabolites in the regulation of signaling pathways related to tumorigenesis.

## Metabolic Characteristics of Pancreatic Cancer and the Tumor Microenvironment

Carbohydrates, amino acids and lipids are used by cells to maintain energy balance and support biosynthesis. In normal cells, glucose is metabolized to pyruvate through glycolysis. Pyruvate enters the mitochondrial tricarboxylic acid (TCA) cycle with the assistance of pyruvate transporter, and then is completely oxidized to carbon dioxide and water through the process of oxidative phosphorylation, producing a large amount of ATP to meet the needs of cell metabolism. In tumor cells, glucose generates pyruvate *via* glycolysis. Subsequently, pyruvate no longer enters the TCA cycle, but is converted into lactic acid under the action of lactate dehydrogenase (LDH). Therefore, the glycolytic pathway of tumor cells will not be coupled with mitochondrial oxidative phosphorylation. Other metabolic characteristics of tumors include imbalanced amino acid uptake, increased nitrogen demand, changes in nutrient acquisition patterns, increased glycolysis/TCA cycle intermediates for biosynthesis and nicotinamide adenine dinucleotide phosphate production, metabolite-driven gene regulation changes, and enhanced microenvironmental metabolic interactions (9). Although metabolism remodeling is a general characteristic of cancer, different cancers show distinct metabolic addictions, which are mainly determined multiple factors such as their specific genetic mutations or tumor microenvironment. Cancer cells exhibit extraordinary growth advantages mainly in three ways (1): Reprogramming intracellular energy metabolism of nutrients (2). Improving nutrient acquisition by scavenging and recycling (3). Conducting metabolic crosstalk with stromal cells within the microenvironment (10). At present, the mechanism of tumor aerobic glycolysis is not clear, it is generally believed to be related to hypoxia and abnormal tumor gene signals. Studies have pointed out that glucose transporters (GLUTs), a family of proteins on the cell membrane that can transfer glucose into cells, can be significantly upregulated in tumor cells, which accelerates aerobic glycolysis and maintain the proliferative advantage of tumor cells (11). In addition, specific oncogenes such as murine sarcoma virus oncogene (KRAS), phosphatidylinositol 3-kinase (PI3K), c-MYC, and hypoxia-inducible factor 1 (HIF1) can also play the same role by regulating pyruvate kinase M2 (PKM2), hexokinase 2 (HK2), and other crucial enzymes in the glycolytic pathway (12). There is also an interaction between tumor metabolic remodeling and Myc. On the one hand, Myc regulate the glycolytic process of tumor cells by activating glycolysis-related proteins such as HK2, glyceraldehyde-3-phosphate dehydrogenase (GAPDH), and enolase-1. On the other hand, changes in tumor metabolic status also activate mammalian target of rapamycin complex 1 (mTORC1) and increase the translation levels of Myc by targeting ribosomal S6 protein kinase (S6K1), thereby forming a positive feedback loop (13). The oncogene KRAS is nearly universally mutated in pancreatic cancer. Oncogenic Kras signaling promotes extracellular glucose avidity and capture *via* upregulation of GLUT1 and HK, respectively. Oncogenic Kras diverts glucose flux into the hexosamine biosynthetic pathway to enhance the generation of precursor moieties required for protein glycosylation. Oncogenic Kras activity also leads to enhanced entry of glucose carbon into the pentose phosphate pathway by which proliferating cells make ribose 5-phosphate (R5P) for DNA and RNA biosynthesis (14). Knock down of Kras-regulated enzymes that govern pentose phosphate pathway is strongly growth inhibitory (15). Tumor suppressor genes have an opposite role in tumor metabolic remodeling. P53 can block the expression of GLUT1, GLUT3, and GLUT4 by interfering with nuclear factor kappa-B kinase α/β (IκBk α/β). The gene promoters of critical enzymes in the aerobic glycolysis pathway, such as *HK2*, contain p53 binding sites; thus, they can also be inhibited by p53 (16, 17). Therefore, deletion of *TP53* gene in tumor cells will promote aerobic glycolysis.

Cancer-associated fibroblasts are the most important component of stromal cells in pancreatic cancer and are in direct or indirect contact with tumor cells. Our research shows that as an important interstitial component of the pancreatic cancer microenvironment, pancreatic stellate cells have a positive feedback relationship with pancreatic cancer cells. Activated stellate cells promote the progression of malignant biological behavior and chemotherapy resistance of pancreatic cancer (18–20). CAFs are continuously activated in the tumor microenvironment. Compared with normal fibroblasts, CAFs have also undergone significant changes in carbohydrate metabolism, similar to the Warburg effect of tumor cells. Studies have shown that as the rate-limiting enzyme of the TCA cycle in the mitochondria, isocitrate dehydrogenase 3α (IDH3α) plays a crucial regulatory role in the aerobic glycolysis of CAFs. Conversely, IDH3α allosterically regulates the activity of proline hydroxylase 2 (PHD2) by adjusting the ratio of α-ketoglutarate (α-KG) to fumaric acid and succinic acid, resulting in the inhibition of PHD2 activity and HIF1α stabilization. The accumulation of HIF1α can strengthen the cell’s aerobic glycolysis process and inhibit the level of oxidative phosphorylation (21). However, carbohydrates produced by aerobic glycolysis in CAFs are not used for cell biosynthesis. Some studies have shown that lactic acid and ketone bodies produced by aerobic glycolysis are exported to adjacent tumor tissues, thereby promoting tumor cell proliferation. In CAFs, the expression of monocarboxylic acid transporter-4 (MCT-4), which exports lactate to the ECM, is upregulated. At the same time, the expression of monocarboxylic acid transporter-1 (MCT-1) on the cytoplasmic membrane of tumor cells is increased, and the metabolites of CAFs are absorbed into tumor cells, indicating that the metabolites exported by CAFs could provide materials for tumor proliferation (22). After CAF metabolic reprogramming, the content of anaerobic metabolism-related enzymes increases significantly, especially the critical rate-limiting enzyme PKM2 in anaerobic metabolism. Studies have shown that overexpression of PKM2 in CAFs can induce larger breast cancer masses in mouse models (23). Glutamine synthetase is the most important enzyme for glutamyl synthesis. Tumor cells can promote the expression of glutamine synthase in CAFs, which is beneficial to mitochondrial metabolism of tumor cells. Glutamine can enhance the autophagy of mitochondria in CAFs, reduce the autophagy of mitochondria in tumor cells, upregulate the expression of glutamine transporters in tumor cells, and enhance mitochondrial biosynthesis in tumor cells (24).

## Interaction Between Tumor Metabolic Remodeling and the Tumor Microenvironment

Pancreatic cancer not only responds to the tumor microenvironment, but also affects the metabolism of stromal cells in the microenvironment. Tumor-derived exosomes can mediate communication between tumor cells and their microenvironment (25). The vesicles of cancer cells can inhibit glucose uptake by other cells in the metastatic tumor, such as fibroblasts and astrocytes, thereby allowing metastatic cancer cells to preferentially take up glucose. These vesicles contain high levels of *miR-122*, which can inhibit the uptake of glucose by stromal cells *via* downregulating glycolytic enzymes such as pyruvate kinase (26). These findings indicate that metabolism remodeling of stromal cells in the tumor microenvironment is a metabolic adaptation process for tumor cells to facilitate their own proliferation.

Tumor cells can choose different metabolic methods to generate ATP and biological macromolecular substances for their own use according to the content and concentration of nutrients such as glucose, glutamine, or fatty acids in the surrounding environment. Studies have shown that metastatic colorectal cancer cells can affect liver cell metabolites to promote colonization of metastatic tumor cells and the formation of liver metastases. The metastatic tumor cells release brain-type creatine kinase to promote creatine phosphate production, which then enters the metastatic colorectal cancer cells to produce ATP. Under the nutritional pressure of lack of glucose or glutamine, tumor cells activate the oncogene c-Myc, metabolize enzyme expression by regulating the serine synthesis pathway molecules such as PHGDH, PSAT1, and PSPH, use the remaining glutamine or glucose to support the *de novo* serine synthesis pathway, and support tumor cell survival under nutritional stress by maintaining redox homeostasis (27). In tumors with *MYC* gene mutations, the expression of the monocarboxylic acid transporter MCT1 and LDH is significantly increased, which promotes the transport and reuse of lactate. In addition, under serum starvation conditions, tumor cells can activate the mTORC2–AKT–SP1 signaling pathway and upregulate the expression of the rate-limiting enzyme 3-ketoacyl-CoA transferase 1 (OXCT1) of ketone body catabolism. Metabolites produced by ketone body catabolism enter the TCA cycle to provide ATP for tumor cells (28). Under hypoxic or nutritional stress conditions, tumor cells ingest acetoacetate to produce acetyl-CoA, which provides energy and biological macromolecules for their survival. During pancreatic cancer progression, there is a hypoxic inner area and an oxygen-rich outer area. Lactic acid can be produced, transported, and effectively used between the two areas. The glycolysis of pancreatic cancer cells in the hypoxic zone produces lactic acid and hydrogen ions that are excreted into the tumor microenvironment through monocarboxylic acid transporter 4 (MCT4), and then are taken up by cancer cells in the peripheral oxygen-rich zone that express MCT1. The glycolysis of pancreatic cancer cells in the hypoxic zone produces lactic acid and hydrogen ions that are excreted into the tumor microenvironment through MCT4, and then are taken up by pancreatic cancer cells in the peripheral oxygen-rich zone that expresses MCT1. Lactate dehydrogenase is reduced to pyruvate NADH, which enters the TCA cycle and becomes the fuel for respiration. After inhibiting the expression of MCT1, peripheral cancer cells die of glucose starvation due to the preferential use of lactic acid for oxidative metabolism. The remaining cancer cells are sensitive to radiotherapy, which suggests an effective combination therapy strategy to tumor treatment (29, 30). Therefore, cancer cells can use the metabolites produced in the microenvironment to cope with the metabolic stress encountered at different metastatic sites. The metabolic status of pancreatic cancer cells is not only the result of their own long-term adaptation, but also affects the fate of surrounding cells, such as cancer-related fibroblasts, endothelial cells, and immune cells. As the tumor grows, these cells undergo a series of metabolic remodeling that leads to phenotypic changes.

### Tumor Metabolism and the Inflammatory Microenvironment

Proper inflammatory response in the body can stimulate the body to improve immunity, but long-term stimulation of inflammatory mediators forms a suitable soil for tumor cell proliferation, that is, the inflammatory microenvironment. Studies have confirmed that chronic inflammation is related to tumorigenesis. Tumors are often accompanied by diseases such as gastritis, gastric ulcer (*Helicobacter pylori*) and gastric cancer, chronic cervicitis (papilloma virus), and cervical cancer. Recent studies have found that some metabolic diseases are closely related to the occurrence of tumors, such as obesity, diabetes, and non-alcoholic fatty liver. The carcinogenic pathway is most likely through the inflammatory response. In 2006, Hotamisligil first proposed the concept of the “metabolic inflammatory response”, providing new ideas for studying the relationship between metabolism and the inflammatory response (31). Pancreatic cancer is a highly metabolic disease, in which many inflammatory factors including cytokines, chemokines, and other inflammatory response mediators participate. Inflammatory mediators have a regulatory role in the synthesis, secretion, and metabolism of nutrients such as glucose, fat, and protein in tumor cells. Researchers have studied the relationship between tumor metabolism and the inflammatory response, and paved the way for clinical diagnosis and treatment (**Table 1**).

**Table 1:** Inflammatory factor that regulating the metabolic remodeling and cancer microenvironment.

Inflammatory factor	Signaling pathway	Effect of metabolic process	Role in immune response	Role in cancer progress	Refs.
Interleukin family	STAT3; mTOR; AMPK	Inhibit cholesterol hydroxylase; promoting glycolysis and glutamine metabolism;	Induces immune inflammatory response; anti-tumor immune response	Promote occurrence and development of cancer	(32–34)
INF-γ	JAK/STAT3; PD-L1; (PI3K)/AKT	Induce inflammatory and catabolic response	Mediates Th1 type inflammatory response	Pro-apoptotic activity	(35)
Hypoxia inducible factor-1	VEGF; ET-1; PDGF; GLUT;	Accelerate the efficiency of glucose metabolism	Suppresses antitumor immune responses	Induce tumor angiogenesis; promote tumor growth;	(36–40)
NLRP3	IL-1β; IL-8; IL-18 IL-33; mTORC1	Glucose metabolism, and amino acid metabolism	Promotes inflammatory responses	Promotes cancer progression and metastasis	(41, 42)
CTRP	PI3K-Akt; TNF-α; IL-1β; IL6/STAT3; Akt/NF-κB	Enhance insulin sensitivity; inhibits gluconeogenesis	Links metabolism, inflammation, and immunity	Promotes tumor cell survival and resistance to chemotherapy-induced apoptosis	(43, 44)

The interleukin family is a widely studied inflammatory cytokine that plays an important role in information transmission and regulation of immune cells. IL-1 (lymphocyte stimulating factor), with two structures (IL-1α and IL-1β), is the main inducer of the immune inflammatory response. Many studies have found that IL-1 is closely related to the occurrence and development of pancreatic, gastric, liver, and breast cancer, amongst others. IL-1 induces LIF expression and downstream JAK/STAT activation to generate inflammatory CAFs in pancreatic cancer, thus promoting cancer progression, chemoresistance and other cancer-associated systemic effects, such as cachexia and immune suppression (45). IL-1β is mainly present in the blood circulation and functions within a cascade of cytokines that initiates the inflammatory response and promote the migration of cancer cells. IL-1 may also have an effect on the anti-tumor immune response (46). Studies have found that inflammatory response mediators participate in regulating lipid metabolism. IL-1, IL-6, and TNF-α can inhibit cholesterol hydroxylase. Studies have shown that IL-4 increases occurrence and enhances metabolism of tumors by promoting glycolysis and glutamine metabolism (47, 48). Lactate dehydrogenase A (LDH-A) is one of the critical enzymes in the glucose metabolism pathway, and IL-4 can upregulate the expression of the glucose metabolism-related gene *LDHA*, thereby promoting the proliferation of tumor cells. IL-6 has a role in regulating metabolic balance and anti-inflammatory responses in obesity-related inflammatory reactions and metabolic diseases. Studies have found that there is a direct regulatory relationship between IL-6 and insulin, metabolic pathways, and inflammatory response signals. IL-6 is overexpressed in a variety of cancers, and it can activate the STAT3 signaling pathway to promote tumor occurrence (32). Lesina et al. found that IL-6 was mainly involved in the JAK/STAT pathway activation promoting acute and chronic pancreatitis disease aggravation as well as pancreatic cancer initiation and progression (33). However, some other studies found that IL-6 regulates tumor metabolism and inflammatory response disorders by inhibiting the mTOR pathway through the activation of AMPK rather than STAT signals (34).

Interferon γ (INF-γ) is an important member of the interferon family with broad-spectrum anti-viral, anti-proliferative, and immunomodulatory activities. INF-γ mainly induces the production of cytokines such as TNF-α and IL-6 to mediate the Th1 type inflammatory response. The mutual influence of these inflammatory response factors can form a vicious circle, which is the main mechanism leading to a sustained inflammatory response (35). The INF-γ-inducing genes (including *CXCL9*, *CXCL10*, and *CXCL11*) encode ligands of chemokine receptor 3 (CXCR3). INF-γ not only induces binding to its receptor to clear cells, but also induces the inflammatory response by recruiting inflammatory effector cells. As one of major cytokine involved in cachexia, INF-γ demonstrates an antiproliferative and antifibrotic capacity, which modulate local anti-tumor immune response. Weight loss in cancer was associated with INF-γ production and administration of an anti-INF-γ antibody reduced the depletion of body fat (49).

Hypoxia-inducible factor-1 is a transcriptionally active nuclear protein with a broad spectrum of target genes, including nearly a hundred target genes related to the development of the inflammatory response, tumor growth, and hypoxia adaptation. Target genes regulated by HIF-1 include vascular endothelial growth factor (*VEGF*), endothelin-1 (*EDN1*), insulin-like growth factor 2 (*IGF2*), and platelet-derived growth factor (*PDGF*). Under the action of these genes, HIF-1 has biological effects such as erythropoiesis, angiogenesis, energy metabolism of amino acids and glucose, cell survival, apoptosis, and drug resistance (50, 51). HIF-1α makes cancer cells resistant to cisplatin, oxaliplatin, and paclitaxel. The hypoxic microenvironment of pancreatic tumors stabilizes HIF-1α, which promotes glucose metabolism. Shukla et al. found that HIF-1α regulated the metabolic phenotype and gemcitabine resistance in pancreatic cancer. Gemcitabine-resistant pancreatic cancer cells increased expression of HIF-1α by upregulating MUC1 expression, along with increased glycolytic phenotype and dependence on glucose (52). Other studies show that HIF-1α can accelerate the efficiency of glucose metabolism and provide the energy needs for cancer cells by regulating the activity of GLUT1 and the transcription of *GLUT1* mRNA (36, 37). The roles of HIF-1α in lipid metabolism reprogramming in cancer is under-studied. Existing evidences show that HIF-1α, promotes fatty acid uptake through induction of FABPs (FABP3, FABP7, and FABP4) along with PPARγ, and lipid storage by modulating ADRP, AGPAT2, and LIPIN1 expressions. Seo and colleagues determined that the FABP5/HIF-1α axis regulates lipid metabolism and cell proliferation in hepatocellular carcinoma (38). HIF-1α can induce vascular target genes, especially vascular endothelial growth factor (VEGF), and induce tumor angiogenesis (39, 40). However, VEGF also regulates HIF1α expression and activation, forming a positive feedback loop between HIF-1α and VEGF. Shi et al. indicated that VEGF enhanced glycolysis by neuropilin 1 (NRP1)-mediated up-regulation of HIF1α and its targeted glycolytic enzymes (53).

NLRP3 is a multi-protein complex mainly expressed in neutrophils and macrophages. The main function of NLRP3 is to activate caspase-1 to indirectly regulate the secretion of interleukin 1β (IL-1β), IL-8, and IL-33. NLRP3 is also identified as a regulator to controls platelet activation and aggregation. Boone et al. reported the NLRP3 inflammasome was upregulated in a murine model pancreatic cancer and promoted platelet aggregation and tumor growth. Pharmacological inhibition of NLRP3 in platelets resulted in decreased platelet activation and improved survival of tumor-bearing mice (54). Studies have shown that the activation of NLRP3 is related to many factors. The possible mechanisms include potassium efflux, oxidized mitochondrial DNA release, mitochondrial dysfunction and reactive oxygen species (ROS) production, cathepsin B release caused by lysosome destruction, changes in intracellular calcium concentration, and transmembrane hole formation (41). NLRP3 inflammasome has an important role in linking metabolism and inflammation. For example, glycolysis is related to the NLRP3 inflammasome through different metabolites. Intermediates or metabolites in the TCA cycle may also be involved in the regulation of NLRP3. The glycolytic enzyme hexokinase-1 (HK1) directly interacts and activates the NLRP3 inflammasome in the outer mitochondrial membrane. In this process, mTORC1 regulates HK1-dependent glycolysis through Raptor’s influence on HK1 expression. Raptor is a regulatory-related protein of the mTORC1 complex and is related to the activation of NLRP3 inflammasomes. Moreover, this activation may in turn promote the expression of HK1 (42). In addition to glucose metabolism, its role in amino acid metabolism is attracting increasing attention and may become a future research hotspot. In addition, NLRP3 inflammasomes are also activated in many diseases, including infections, autoimmune diseases, and various cancers such as stomach, colorectal, liver, lung, and cervical cancer. The main mechanism is mainly related to the activation of IL-1β and IL-6 signaling pathways (41).

Complement-C1q/TNF-related protein (CTRP) is a newly discovered adipokine superfamily, and 15 members have been discovered so far. CTRP contains an amino-terminal signal peptide, a short variable domain, a collagen-like domain, and a carboxy-terminal spherical domain. Recent studies have shown that CTRP family members participate in the regulation of glucose and lipid metabolism and inflammation. CTRP12 is an adipose factor, and it is secreted by adipose tissue that can enhance insulin sensitivity, improve insulin resistance, and reduce the inflammatory response of adipose tissue. CTRP12 inhibits liver gluconeogenesis and adipocyte glucose uptake by activating the PI3K–AKT signaling pathway. CTRP3 also has similar characteristics. It inhibits gluconeogenesis by downregulating the expression of the rate-limiting enzymes glucose 6-phosphatase and phosphoenolpyruvate carboxykinase, which can also improve the insulin sensitivity of adipocytes and promote the expression of adipokines adiponectin, leptin, and visfatin. CTRP6 expression is significantly increased in the state of high glucose. CTRP6 stimulates the generation of ROS, induces inflammation and ECM accumulation by upregulating the expression levels of tumor necrosis factor-α (TNF-α), IL-1β, IL-6, and the AKT–NF-κB pathway (43). CTRP4 promotes tumor cell survival and resistance to chemotherapy by effectively inducing the activation of the NF-κB and IL6–STAT3 signaling pathways (44).

Other inflammatory response mediators related to tumor metabolism disorders include proteolysis-inducing factor (PIF), which stimulates and activates the NF-κB pathway, thereby inducing the secretion of a variety of inflammatory factors, such as IL-6/IL-8 and ICAM-1 (55). Lipid-mobilizing factor (LMF) can promote the decomposition of fat tissue under the action of β3 adrenal receptors. Other cytokines include leukemia inhibitory factor (LIF) and ciliary neurotrophic factor (CNTF). The former can inhibit the activity of lipase and promote lipolysis, while the latter is related to the metabolic disorders of fat and protein. Neutrophil gelatinase-associated lipocalin (NGAL) is a member of the lipocalin family of proteins. NGAL is involved in cell glucose and lipid metabolism and energy regulation. At the same time, NGAL can form a complex with matrix metalloproteinase 9 (MMP9). This MMP9/NGAL complex is related to tumor proliferation, metastasis, and chemotherapy resistance (56).

### Pancreatic Cancer Metabolism and the Acid Microenvironment

The metabolic remodeling of pancreatic cancer is an important reason for the formation of an acidic microenvironment. Low energy but rapid energy supply method generates heat and leads to an increase in the production of lactic acid, thereby inducing the production of an acidic tumor environment (57). Another major feature of the pancreatic cancer microenvironment is hypoxia. Hypoxia is caused by the rapid proliferation of cancer cells. The hypoxic environment of pancreatic cancer cells can activate HIF. In this environment, HIF-1 induces the expression of carbonic anhydrase IX (CA IX) and CA XI to facilitate extracellular acidification. HIF contributes to the formation of glycolytic phenotypes in cancer cells, which is acidified by lactic acid production. HIF converts pyruvate into lactic acid by upregulating lactate dehydrogenase-A (LDH-A) and directly upregulates the expression of GLUT1 and GLUT3. In summary, HIF plays a central role in the regulation of energy metabolism. It promotes the production of lactic acid and forms the acidic microenvironment by converting mitochondrial oxidative phosphorylation to anaerobic glycolysis under hypoxia (58). The acidic tumor microenvironment can induce the expression of MMPs such as MMP-2 and MMP-9 and enhance the invasion and metastatic ability of tumor cells. Cancer cells exposed to acidosis have the characteristics of an epithelial–mesenchymal transition phenotype, as well as high invasiveness, high anti-apoptotic ability, and anti-drug therapy properties (59). Moreover, aberrant glycolysis is also a promoting factor for tolerance to chemotherapeutic drugs. 2-DG (a synthetic glucose analog)-induced glycolysis inhibition markedly improves pancreatic cancer sensitivity to gemcitabine (60).

CO_2_ produced in the process of tumor fatty acid metabolism is hydrated to HCO_3_^−^ and H^+^ by CAs. The process of fatty acid synthesis palmitate in tumor cells produces CO_2_ and H^+^. To avoid intracellular acidosis and maintain pH homeostasis, tumor cells increase the expression of transporters and channels to promote H^+^ excretion. The main acidic metabolites accumulated in cells are lactic acid and hydrogen ions, which are mainly excreted by MCT1 and MCT4. The hydrogen protons produced in the cell are also discharged outside the cell by Na+/H+ exchangers (NHE), which causes acidification outside the cell. CA IX and CA XII are transmembrane CAs with extracellular catalytic domains that catalyze the hydration of extracellular CO_2_ to generate HCO_3_^−^ and H^+^. Lack of sufficient functional blood vessels is also a common feature of pancreatic cancer that affect the acidification of tumors. The lack of vasculature reduces the oxygen supply and the removal of acidic waste, leading to the accumulation of H^+^ in the poorly perfused microenvironment. Therefore, the increase in lactic acid and CO_2_ caused by abnormal tumor metabolism, hypoxia, and poor perfusion are the main reasons for the formation of an acidic tumor microenvironment. Ion transporters and CAs can reverse the acidic microenvironment of pancreatic cancer to a certain extent by targeted regulation of the pH gradient of the microenvironment, thereby inhibiting tumor cell proliferation and reducing tumor drug resistance. A study showed that the combined use of proton pump inhibitors and CA inhibitors achieved more effective anti-tumor effects than single-drug therapy (61). The development of drugs that reverse the pH gradient of the tumor microenvironment can provide new strategies for effective anti-cancer treatments.

### Tumor Metabolism and the Immunosuppressive Microenvironment

In the process of pancreatic cancer development, in addition to obtaining the nutrients needed for their rapid proliferation, it is also necessary to escape the attack from immune system. Lactic acid is the final product of glycolysis in tumor cells. The effects of extracellular lactic acid include: (1) preventing the transformation of monocytes into dendritic cells; (2) inhibiting the release of cytokines from dendritic cells and cytotoxic T cells; (3) inhibiting monocyte migration; and (4) decreasing the function of cytotoxic T cells. Pancreatic cancer cells can inhibit the activity of cytotoxic T cells and antigen-presenting cells by controlling the acidity of the tumor microenvironment and further increasing the glycolysis of tumor cells, leading to the immune escape of tumor cells (57).

**Figure 1:**
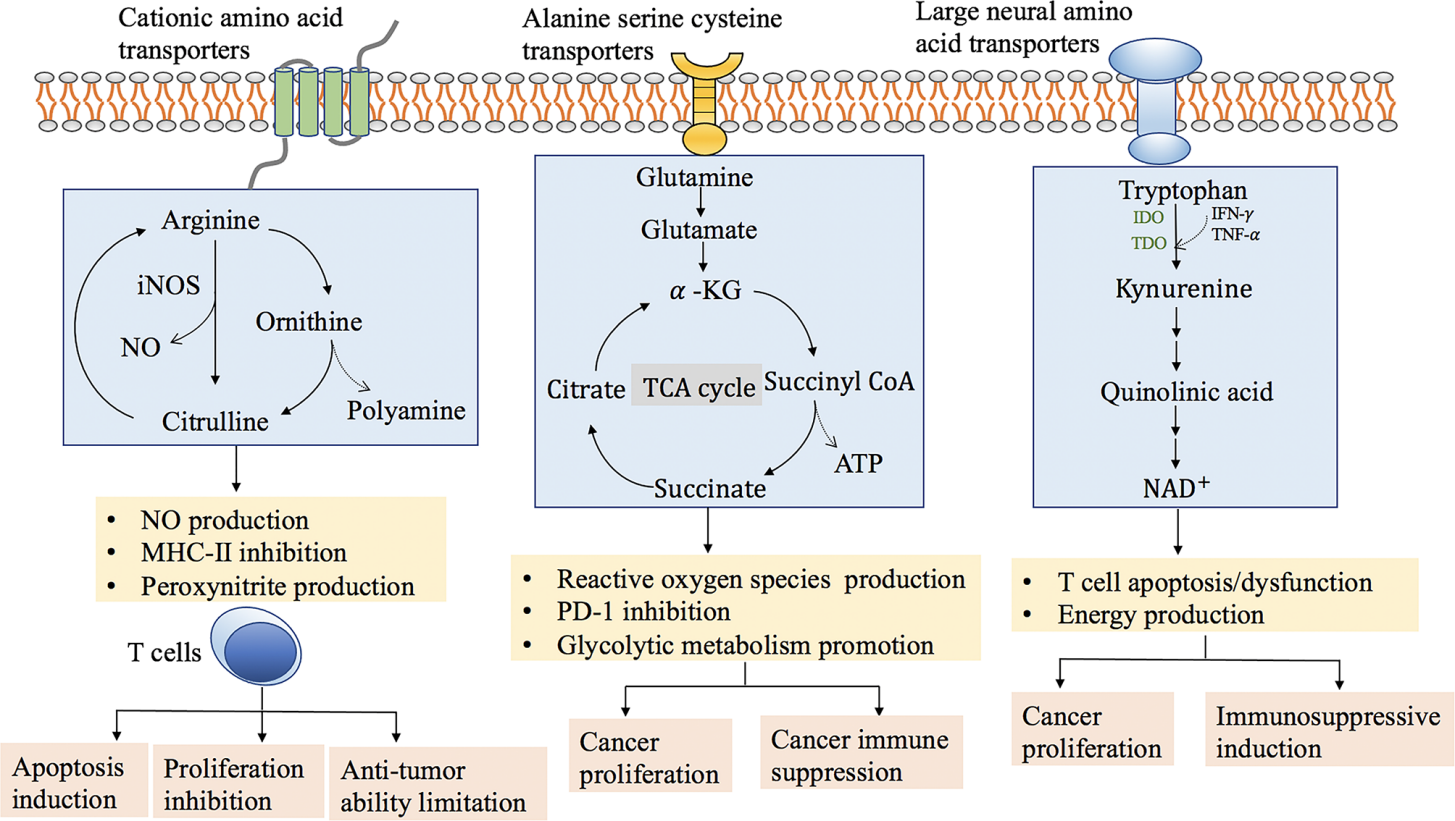
The role of amino acids, arginine, glutamine and tryptophan metabolism in cancer progression and immune function.

The metabolic level of amino acids in pancreatic cancer cells changes to adapt to the increase in energy demand. Changes in the amino acid metabolism pathways of pancreatic cancer cells are often driven by multiple signaling pathways and transcription factors (**Figure 1**). A large number of basic research studies and clinical trials have shown that development of new drugs for regulation of tumor-dependent amino acid metabolism can effectively inhibit tumor growth.

Arginine metabolism is an important mechanism to regulate the responsiveness of immune cells. Arginine and its downstream metabolites (such as ornithine and citrulline) may be essential for T cell activation, thereby regulating innate and adaptive immunity (62). Decreased arginine content in tumors can inhibit the function of T cells, especially CD8^+^ T cells. Both myeloid-derived suppressor cells (MDSCs) and macrophages in the tumor microenvironment can induce the expression of arginase (ARG) and nitric oxide synthase (NOS). ARG degrades arginine into ornithine and urea, and NOS oxidizes arginine to citrulline and nitric oxide (NO). NO inhibits the proliferation of T cells by inhibiting the expression of major histocompatibility complex II (MHC-II). Tumor-derived cytokines such as transforming growth factor-β (TGF-β) can induce ROS production in MDSCs. The peroxide molecule (O_2_^−^) reacts with NO to produce peroxynitrite (PNT). PNT can induce the apoptosis of T lymphocytes, and also nitrate and nitrosylate T cell receptors and CD8 molecules, rendering T cells resistant to tumor cells. The nitrosylated T cell receptor loses its ability to recognize specific peptides and MHC complexes, thus limiting the anti-tumor ability of CD8^+^ T cells.

Tryptophan is necessary for T cell division and proliferation. In the absence of tryptophan and tryptophan breakdown products, activated T cells are stagnant in S phase, unable to synthesize DNA, and are extremely sensitive to Fas-mediated apoptosis, which indicates that the reduction in tryptophan can lead to immunosuppression. Indoleamine2,3-dioxygenase (IDO) is a tryptophan-decomposing enzyme overexpressed in melanoma, colon cancer, and renal cell carcinoma, and is closely related to the prognosis of tumors (63). In the tumor microenvironment, IDO can be secreted by tumor cells, tumor-associated macrophages, and regulatory T cells. IDO is highly expressed in macrophages and dendritic cells, and directly inhibits T cell functions, thereby making the tumor microenvironment an immune tolerance environment. Except for IDO1, tryptophan catabolism by tryptophan-2,3-dioxygenase (TDO2) is a feature of many tumors, especially malignant gliomas (64, 65). The accumulation of metabolites caused by increased tryptophan metabolism, such as 3-hydroxykynurenine and 3-hydroxyanthranilic acid (3-HAA), can trigger immunosuppression. Kynurenine binds to aryl hydrocarbon receptor (AHR) to inhibit T cell activity. Decreases in tryptophan and tryptophan metabolites lead to downregulation of CD8^+^ T cell receptor ζ chain and inhibit CD8^+^ T cell expansion *in vitro*. The catabolism of the essential amino acid tryptophan is a crucial metabolic pathway for the formation of the immunosuppressive tumor microenvironment, and thus it is a feasible drug target for tumor immunotherapy.

Glutamine is an important cancer cells metabolism substrate. Pancreatic cancer cells grown in culture are strictly dependent on glutamine for proliferation. Glutamine facilitates generation of reducing equivalents in the form of NADPH is driven by oncogenic Kras. Kras activates the GOT2-GOT1-ME1 pathway and initiates a nuclear factor (erythroid-derived 2)-like 2 (Nrf2)-dependent reactive oxygen species (ROS) detoxification program. Mutant Kras constitutively activates this antioxidant program to suppress ROS and enhance pancreatic tumorigenesis (66). Glutamine can be converted into *α*-ketoglutarate (*α*-KG) through following mechanisms; either by glutamate dehydro- genase (GLUD1) or transaminases. Many cancer cells rely on GLUD1-mediated glutamine conversion. However, being different from other cancer models, pancreatic cancer cells metabolize glutamine in a manner that transaminases is critical for glutamine metabolism (67). Glutamine deficiencies in pancreatic cancer can modulate adaptation mechanisms through signal transduction. Recouvreux et al. reported that glutamine depletion increased Slug expression to promote epithelial–mesenchymal transition (EMT) and metastasis (68). Glutamine is required to support optimal lymphocyte proliferation and pro- duction of cytokines by lymphocytes and macrophages. Research found that tumor-specific CD8^+^ T cells cultured under glutamine-restricted (dGln) conditions or adoptive transfer of CD8^+^ T cells treated with specific inhibitors of glutamine metabolism can effectively eliminate tumors. In addition, PD-1 expression on tumor-infiltrating CD8^+^ T cells cultured with dGln was downregulated, and the positive rate of Ki67 was increased, indicating that inhibition of glutamine metabolism can prevent CD8^+^ T cells from failing *in vivo* (69). The use of glutamine antagonists can destroy the tumor’s metabolic immune suppression microenvironment. There is evidence that glutamine blockade in tumor-bearing mice inhibit the oxidation and glycolytic metabolism of cancer cells. In contrast, the response of effector T cells to glutamine antagonism is to significantly upregulate oxidative metabolism and extend cell lifespan (70). Glutamine antagonism reveals a metabolic interaction between tumor cells and effector T cells that can be used as a “metabolic checkpoint” for tumor immunotherapy.

## Conclusions

Selectively targeted tumor cell metabolic remodeling is an attractive direction for tumor therapy. However, this method has many problems. Because the enzymes in the metabolic pathway often have multiple subtypes, small molecule inhibitors may be unable to distinguish the subtypes of metabolic enzymes expressed in tumor cells from normal cells. Even if specific inhibitors are developed, tumor metabolism is heterogeneous and highly adaptable, and tumors will develop another metabolic pathway. Therefore, to avoid the adaptive resistance of tumor cells, combination of two metabolic pathway inhibitors can be used, and the metabolic inhibitor can also be tried as an auxiliary treatment plan for other treatment methods.

The metabolic pathways adopted by tumor cells are diverse and heterogeneous, and there is also a metabolic symbiosis with stromal cells in the tumor microenvironment. In addition to aerobic glycolysis, metastatic tumor cells can also adopt complementary metabolic pathways such as oxidative phosphorylation and enable them to quickly adapt to new metabolic needs. This metabolic flexibility limits the effectiveness of single targeted therapy. Metabolism targeted therapy is not yet recommended as regular treatment in most guidelines for treating cancers. Our future challenge is to make a deeper understanding of how tumor cells maximize the use of the surrounding resources to maintain survival by influencing the tumor microenvironment (CAFs, macrophages, fat cells, et al). Understanding the mechanism of tumor metabolic adaptation and the metabolic-dependent symbiosis between tumor cells and the surrounding microenvironment may provide a new approach for tumor treatment. This is of great significance for guiding tumor metabolism research, accelerating the development of targeted tumor metabolism drugs, and developing individualized tumor metabolism treatments.

## Author Contributions

YL: Original draft preparation and information collection. JZ: Acquisition of data and revision the manuscript. JX: Interpretation of data and analysis of data. SL: Conception and design of study. All authors contributed to the article and approved the submitted version.

## Funding

The study was supported by the National Natural Science Foundation of China (Grant No.81802888) and the Key Research and Development Project of Shandong Province (Grant No.2018GSF118206; No.2018GSF118088).

## Conflict of Interest

The authors declare that the research was conducted in the absence of any commercial or financial relationships that could be construed as a potential conflict of interest.

## Publisher’s Note

All claims expressed in this article are solely those of the authors and do not necessarily represent those of their affiliated organizations, or those of the publisher, the editors and the reviewers. Any product that may be evaluated in this article, or claim that may be made by its manufacturer, is not guaranteed or endorsed by the publisher.
